# Redefining the role of the transfusion medicine physician in the era of advanced cellular therapies

**DOI:** 10.1111/trf.70168

**Published:** 2026-03-11

**Authors:** Eric A. Gehrie, Kevin J. Land

**Affiliations:** ^1^ New York Blood Center Enterprises Rye New York USA; ^2^ Vitalant Tempe Arizona USA

While the transfusion medicine physician has historically focused on the prevention of serious adverse events during the collection or transfusion of blood, rapid growth in cellular therapy collections and cellular processing has revealed the need for a greatly expanded role. This new persona, which includes overseeing complex laboratory and apheresis operations, providing clinical consultations, directly interacting with the treating team, securing resources and partnerships to accommodate a large number of research protocols, and providing expert insight into how best to solve or interpret complex patient diagnostic issues is necessary to ensure that cellular therapies are as available, safe, and effective as traditional allogeneic blood transfusions.^1^, ^2^

Today's transfusion medicine physician may support several hospitals, some of them remotely, with weekly to quarterly in‐person visits. In other areas, oversight of transfusion medicine activities within a hospital may fall under a pathologist primarily focused on anatomic diagnoses. The net result is that too many pathologists are perceived as remote economic gatekeepers who are adjacent to patient care operations, rather than essential members of the patient care team.^2^, ^3^ The emergence of licensed and investigational biotherapies has coincided with an opportunity to reinvent the transfusion medicine physician as an active and indispensable part of patient care (Figure 1).

**FIGURE 1:**
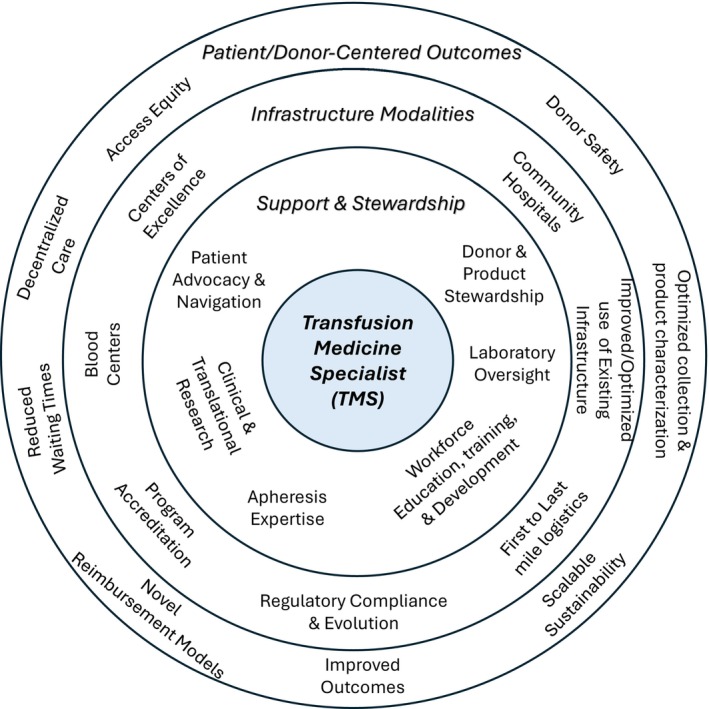
Leveraging the expertise of the transfusion medicine specialist results in improved patient, donor, and programmatic outcomes.

Advanced cell and gene therapies are complex to coordinate, expensive to manufacture and require a high degree of inter‐disciplinary communication and support—comparable to that required for organ transplants. All currently licensed therapies require dedicated collection and manufacturing runs for each patient. While these drugs initially focused on hematological malignancies, they are rapidly expanding into other disease areas, with 4469 therapies in development,^4^ further increasing the demand for apheresis and cell therapy laboratory resources. This growing demand is already beginning to exceed what the traditional university‐hospital model—originally built around hematopoietic stem cell transplantation—can easily accommodate.^5^ This evolving landscape presents an opportunity for physicians to position themselves as facilitators and collaborators who “increase value” by using their expertise to move across the traditional departmental silos, ultimately serving as patient advocates who help ensure access to a rapidly increasing portfolio of advanced therapies.

True sustainability of clinical biotherapies services will require the collective expertise of a broader group of professionals than solely the transfusion medicine physician. Transfusion medicine specialists (TMS)—a term used here to encompass not only physicians but also nurses, clinical laboratory scientists, quality and regulatory experts, advanced practice providers, and other key contributors—are uniquely positioned to support and lead the interdisciplinary efforts necessary to ensure scalable, equitable, and resilient biotherapies infrastructure.

One frequently discussed avenue for increased patient access to cellular therapies is via the collections and laboratory infrastructure of the allogeneic blood collection center. Over the past 10–15 years, a concerted effort to drive cost out of traditional allogeneic blood products, coupled with reduced demand for blood and greater difficulty recruiting volunteer donors, has led to significant consolidation and cost‐cutting. Five community‐based blood centers now collect two thirds of all blood in the United States.^6^ Indeed, standardization of allogeneic blood offerings has established a framework for meeting the current good manufacturing practice (cGMP) goals of Safety, Quality, Identity, Potency, and Purity during manufacturing and safeguarding the Rights of Blood Administration (derived from Rights of Drug Administration), resulting in the right blood going to the right patient at the right dose at the right time for the right reason(s), following the right patient education.^7^, ^8^ It is possible that the application of some of these principles to the collection and manufacture of cellular therapies could greatly improve patient access and potentially diminish associated healthcare costs.

The question that stands before our field is: can the transfusion medicine specialty—which has long focused on standardization, cost‐cutting, and consolidation—pivot to become a critical enabler in the rapid scaling of both research and clinical cellular therapies? Our hypothesis is that TMSs are ideal stakeholders working in concert to navigate this terrain and effectively increase patient access to high‐quality advanced cellular therapies. Some of the challenges that the field faces to address this question are already well established. These include: the need for scalable collection infrastructure; donor safety and product stewardship; the integration of technology and informatics; sustainable funding mechanisms reflecting the true value of cell therapy services (other than the final product) such as apheresis, and the education and training of the next generation of professionals to sustain and advance the field. Each topic is discussed along with a list of questions that remain unanswered. While not exhaustive, the intent is to represent key areas where the TMS can contribute meaningfully—not only by answering them directly, but also by identifying and convening the right collaborators.

## 1 THE FUTURE OF CSM COLLECTION: FLEXIBLE, DECENTRALIZED, AND PATIENT‐CENTERED

Hospitals alone cannot meet the full demand for mononuclear cell collections required to treat the eligible patient population.^9^, ^10^, ^11^, ^12^, ^13^ Although most hospitals are part of a healthcare network, the infrastructure needed to collect cellular starting material (CSM) is typically concentrated in one or two locations. Often, these locations are geographically close to a center of excellence for comprehensive cancer care. A 2024 McKesson survey noted that “for most Americans who don't live near a major academic center, these innovative therapies aren't readily available, creating vast ‘CGT deserts’,”^14^ highlighting both geographic and systemic barriers to access.

One practical way to extend access—particularly for (relatively) stable patients—is to offer care, including apheresis collection, in outpatient settings as close to the patient's community as feasible.^15^ However, most hospital‐based apheresis teams are not mobile, and current patient volumes, combined with workforce limitations, make it impractical for remaining hospitals to build their own clinical apheresis capabilities. Unless these barriers are addressed, they will continue to limit patient access—especially in rural areas—and drive unnecessary costs of care.^15^

Fortunately, there are several ways that the collection of CSM by apheresis could be decentralized. In the end, one needs space; collection, testing, and/or monitoring equipment; a limited pharmacy; experienced staff; back‐office support such as billing, and the appropriate quality management system.^11^ Hospital‐based programs already possess these elements; however, some blood centers also provide these services. By working together, existing hospitals and blood center‐based programs could expand patient access with a hybrid solution using resources from more than one organization. Over the last two editions, the Association for the Advancement of Blood and Biotherapies (AABB) Cell Therapy Standards Committee has worked to ensure sufficient flexibility in the standards, allowing nearly every step of the process to be provided by another party, such as a blood center or another hospital.

Patients could be categorized in several ways, including:

(1) the most critically ill patients being collected at the hospital, where they can benefit from the additional support.

(2) collections for certain therapies consistently being performed by either the hospital or the blood center.

(3) the blood center handling a set number of collections during periods of high demand, or

(4) the blood center supporting collections for patients who prefer or are not able to travel to the main hospital.

For geographies without existing apheresis hospital‐ or blood center‐ based infrastructure, a TMS team could oversee the collection needs of multiple hospitals and clinics within a community or across several communities, a model familiar to the transfusion medicine physician. Qualified and experience apheresis nurses could provide apheresis services in a clinician's office, at a community‐based hospital (including those without large cancer care units), a mobile bus, or at one of the blood center's local fixed sites. All of these settings are challenged by workforce shortages affecting physicians, nurses, and advanced practice providers.

Most blood collection facilities are built for healthy donors. Much less than 1% of blood component therapy is autologous,^6^ whereas 60%–70% of currently approved and late‐stage pipeline CGT products are autologous‐derived. If the trend of treating sicker patients with CGT continues, blood centers will need to significantly reimagine their physical space and operational capabilities, moving beyond models designed primarily for allogeneic donors. This will likely include real‐time laboratory testing, pharmacy and radiology integration, central venous catheter access, and medical support for symptoms like nausea and pain during apheresis. While not feasible everywhere, this model is worth exploring in some locations.

In some academic medical centers (e.g., advanced therapy centers, or simply ATCs), it is not uncommon to encounter a 6–8 week waitlist for collection, with healthier patients at risk of being delayed to prioritize more urgent cases. Prioritizing treatment for the sickest patients is a well understood practice across medicine, and it is important that this principle remain intact. However, it is also important to acknowledge that healthier individuals often have better overall outcomes—not to mention provide CSM that leads to more robust and reliable cellular manufacturing.^16^ Chimeric antigen receptor (CAR) manufacturing failures currently can reach 25% or higher and are linked to low CAR cell activation, poor expansion in vivo, or T‐cell exhaustion.^17^ A recent study showed that early collection of CSM for acute lymphoblastic leukemia patients improved the chances of subsequent successful therapy.^18^ As the demand for more CSM collections increases, including patients who are relatively more stable than late‐stage oncology patients, hospitals and blood centers can work together to optimize the outcome of a successful CSM collection and CAR infusion for all patients.

Disparities to access persist. According to 2024 research, patients living 2 to 4 h from a CAR‐T treatment center were nearly 40% less likely to receive therapy compared to those living within 30 min.^19^ Black patients were less than half as likely to receive CAR‐T as white patients, and patients with more comorbidities were more likely to receive it. Expanding local collection access is key to reducing racial and geographic disparities, promoting earlier therapy initiation and improved clinical outcomes.

While some patients prefer treatment at specialized ATCs, others value staying within their local community—especially when that community has an adequate healthcare infrastructure and qualified providers.^20^ Community‐based apheresis programs, when properly equipped and staffed, offer patients an opportunity to remain close to home for cell collections, reserving travel to distant ATCs for infusion and follow‐up care related to their manufactured therapy. This approach can reduce disruption to daily life and help patients stay connected to their support systems, which are associated with better outcomes.^21^

Leveraging the existing physical and virtual resources of each entity—the clinical infrastructure from the academic medical center, the collection infrastructure from the blood center, and perhaps space and clinical staff to support patients during and after collection from a third‐party community clinical care space (e.g., hospital or clinic)—could feasibly result in scalable, enhanced patient access at minimal cost increment. This type of collaboration may be especially critical for pediatric patients, whose unique clinical requirements often exceed the capabilities of standard adult‐focused hospitals and collection sites.^22^ However, it must also be acknowledged that in the current healthcare environment, many stakeholders and collaborators are already likely working near full operational capacity, and the feasibility of assuming additional workload remains uncertain.

As clinical biotherapies evolve, the traditional models of apheresis collection and the concept of community are being reimagined. A compelling opportunity lies in expanding patient access points to aspects of their care that do not need to be performed at a specialty center.

Questions to consider:

- Can a comprehensive out‐of‐hospital cellular collections model be designed that focuses on equitable patient access and uses existing resources and infrastructures across multiple organizations?
- What regulatory relief would be needed to feasibly operate a network of community‐based collection centers without requiring each center to undertake a full and independent regulatory and accreditation review/certification?
- Is it conceivable that a regulatory framework could be advanced which allowed community‐based collection centers to rapidly expand in number and capacity without unnecessarily increasing patient costs?
- When would it make sense to facilitate collecting more medically complex patients outside of a traditional hospital setting?
- What medications should become routinely part of the CSM collection toolkit to better support the patient—including symptomatic support—before, during, and immediately following apheresis of both stimulated and non‐stimulated collections?
- What role should alternative payment models such as value‐based enterprises (VBEs) have in expanding access to advanced cell and gene therapies?

## 2 IMPROVING THE SAFETY AND EFFICIENCY OF THE APHERESIS PROCESS FOR BOTH ALLOGENEIC AND AUTOLOGOUS DONORS

The traditional blood collection and manufacturing process has certain general thresholds for initiating collection but does not target specific cell subsets. While there have been efforts to connect donor characteristics to product attributes or recipient outcomes,^23^ the vast majority of allogeneic blood from typical donors is suitable for ABO‐compatible recipients. Processes for completing manufacturing and ensuring quality control are therefore simpler, highlighted by the fact that required quality control testing can often be performed on a typical hematology analyzer.

In contrast, cellular therapies are exquisitely sensitive to donor characteristics, often in ways that require extensive donor screening, flow cytometry or molecular‐based product characterization, or both. Donor and product characterizations predict manufacturing success and ultimately impact patient outcomes.^24^ There is much not yet known about who the best allogeneic donor may be for a given cell intended for a given patient.^25^ Despite this, most CSM, regardless of cell subtype ultimately being used for manufacturing (e.g., T‐cell, NK‐cell, dendritic cell, etc.), is currently collected using surrogate parameters and not a direct evaluation of the cell of interest.^26^

Alternatively, the apheresis collection process could be fine‐tuned to the cell of interest if manufacturers shared key product quality data with apheresis sites.^27^ This would enable sites to correlate donor and procedure variables with final product specifications, allowing for continuous process improvement and targeted collections protocols.^28^ While regulatory requirements focus on post‐manufacturing qualification, collection centers rarely receive feedback unless there is a failure attributable to them. Some collection centers have begun sampling pre‐manufacture, but without collaboration, these efforts will have limited benefit. Greater transparency could help optimize collection duration, reduce donor risk, and expand capacity—especially as therapies become more personalized.

Little is known about the long‐term impact of frequent mononuclear cell apheresis procedures on allogenic donors. No standardized, evidence‐based safety criteria have been established, as very few studies have been published on this subject. As a result, donor eligibility and deferral strategies vary widely across the industry.

More is understood about the immediate physiological effects of donation. Table 1, for example, summarizes the changes in peripheral blood white blood cell, hemoglobin, and platelet counts before and after a single donation in 17 stimulated healthy allogeneic donors (5 male, 12 female) collected in November 2024 at one blood center collection site in Tempe, Arizona. As expected, white blood cells and platelets frequently decline immediately following collection. These donors, who support traditional hematopoietic stem cell nationwide, typically undergo apheresis collection only once or twice, and their peripheral counts generally return to baseline within 2–4 weeks. Such data are useful in establishing safety guardrails—helping define how long and how frequently a donor can be collected based on their presenting values, particularly in cases where collections span multiple days.

**TABLE 1:** Changes in peripheral blood white blood cell, hemoglobin, and platelet counts pre‐ and post‐donation in 17 stimulated, healthy, allogeneic donors.

	Metric	WBC	HGB	PLT (K/mL)
Presenting values	Average	51.33	3.35	273.47
	Minimum	21.57	11.70	181.00
	Maximum	94.60	15.40	351.00
Overall change	Average	−6.77	−0.75	−98.85
	Minimum	10.12	0.10	−41.00
	Maximum	−17.30	−1.60	−218.00
Per hour of collection	Average	−2.12	−0.23	−23.98
	Minimum	2.34	0.01	−13.17
	Maximum	−8.33	−0.56	−37.59
Per liter blood processed	Average	−0.88	−0.10	−0.98
	Minimum	1.26	0.00	−5.22
	Maximum	−3.43	−0.22	−13.23

What is the long‐term impact of frequent mononuclear cell apheresis (aka leukopaks, or source leukocytes) on donors? Clients frequently request 6–12 week intervals between donations to allow time for white cell subsets to return to baseline (K Land, personal communication). A recent report identified T‐cell lymphopenia in frequent volunteer apheresis platelet donors.^29^ Notably, lifetime donations of ≥50 were significantly associated with reduced CD4+ and CD8+ counts on the Trima Accel (with leukocyte reduction system [LRS] chambers). The lymphopenia persisted for 1–2 years after donations ceased. Despite low T‐cell counts, the immune function appeared relatively preserved, although there was a signal that mild infections (e.g., herpes zoster) were slightly more common. LRS chambers contains an average of 1.1 ± 0.3 billion leukocytes, with a mean 80.6 ± 13.1% MNCs.^30^

Fresh leukopaks are usually sold in full, half, quarter, and occasionally one‐tenth sizes. A full leukopak contains at least 10 billion white blood cells with approximately 95% MNCs.^31^, ^32^ Thus, a single full leukopak removes the equivalent number of MNCs as ~10 LRS chambers. Extrapolating from the apheresis platelet donor finding, a leukopak donor could be at significant risk of lymphopenia after only 4 or 5 collections. A study in 2012 found no evidence of immune depletion in six healthy adult frequent leukapheresis donors; however, the sample size was small and the total leukocytes removed at each donation were less than a full leukopak.^33^ Clearly, more studies need to be performed to see if this theoretical risk exists, but it does reveal how much still remains to be learned about CGT donor safety.

Interestingly, researchers reported during a plenary session at ASH in 2024 that extensive whole blood donation (>100 lifetime donations) is associated with clonal hematopoiesis, due to selective expansion induced by elevated EPO levels.^34^ The clonal hematopoiesis was seen in specific *DNMT3A* mutations that were different from leukemogenic *DNMT3A* R882 mutations. The authors and commentators noted that this study identifies EPO as a novel environmental factor.^35^ It is unclear at present what type of selection pressures frequent leukapheresis may induce nor what risks they may impose on donors.

The optimization of the collection of CSM by apheresis remains a significant challenge. Although much can be learned by adopting many of the good manufacturing practices in traditional blood collection, the donor characteristics—such as immune cell subset composition, exhaustion markers, and genetic traits beyond HLA genotypes—can dramatically influence manufacturing success and patient outcomes. Yet, most collections are performed using surrogate parameters (e.g., total nuclear cell count, total blood volumes processed, or total hours collected) rather than direct evaluation of the target cell type(s). Proprietary data practices by the cell and gene therapy vendor have in part stifled efforts to correlate donor and collection variables with the final product. As donor characterization becomes increasingly sophisticated—incorporating genetic, proteomic, and molecular profiling—ethical considerations around informed consent and data privacy grow more complex, particularly under frameworks like the Genetic Information Nondiscriminatory Act (GINA), which prohibits the misuse of genetic data but does not fully address the nuances of donor rights in the context of CSM.

Equally concerning is the limited understanding of the long‐term safety for healthy allogeneic donors who undergo frequent leukapheresis. As the industry moves toward increased reliance on allogeneic cell sources, donor safety will become paramount. While immediate post‐donation declines in white blood cells, hemoglobin, and platelets are well‐documented, there is little evidence‐based guidance on cumulative effects. Limited data suggest frequent donations may result in persistent lymphopenia, raising questions about immune resilience and potential long‐term risks. The industry urgently needs longitudinal studies and standardized safety criteria to protect frequent donors. Transfusion medicine physicians have the most applicable skills and expertise to lead these studies. Without these advancements, donor welfare and product consistency and overall availability will remain vulnerable. Once donors lose confidence in the industry, access to allogeneic CSM will dwindle. Instead of relying on a single donor for CSM, the industry will likely need to rely on a pool of well‐characterized, reliable donors derived from a much larger database of interested donors.

Questions to consider:

- Can manufacturers be incentivized to share more manufacturing outcome data with collection centers?
- If so, will manufacturers be receptive to feedback from collection centers regarding methods to improve the collection process?
- Can regulatory relief be obtained for laboratories which do not seek to perform diagnostic testing, but would like to use diagnostic testing in order to perform donor and product characterization and qualification?
- Should there be a lifetime limit on leukopak donations? When should additional testing be included into donor qualification to ensure donor safety, and what testing would be informative in this population?
- What is the potential role for volunteer allogeneic leukapheresis donors, as opposed to paid allogeneic leukapheresis donors?
- As donor profiling expands to include pre‐collection, manufacturing, and outcome‐related data, how should the allogeneic donor consenting framework evolve to address the ethical use of genetic, proteomic, and immunologic data?
- What additional elements will need to be incorporated into pharmacovigilance to capture and monitor acute and chronic adverse events in donors and patients of cell and gene therapies?
- What is the minimal ethical framework needed to begin to use artificial intelligence to analyze the complex datasets that will be generated?

## 3 FROM MANUAL TO MODULAR: EVOLVING CGR THROUGH STANDARDIZATION AND IT INTEGRATION

Anyone who tours a traditional blood component manufacturing laboratory is likely to encounter a plasma expressor device and, nearby, a centrifuge. Both devices have been central to blood component manufacturing for decades. What is striking is that automation is almost entirely absent—each product is typically hand carried throughout the component manufacture laboratory. Importantly, however, all donors are qualified in the same way using (ideally) a blood establishment computer system (BECS). Similarly, products are treated in nearly the exact same way, that is, from initial receipt through labelling and release. BECS is a specialized software system FDA approved to manage and monitor all aspects of blood collection, donor blood loss tracking, testing, inventory, and sometimes even transfusion. It plays a crucial role in ensuring safety, regulatory compliance, and operational efficiency. To ensure quality and consistency and an affordable cost, any successful blood component processing program must be built on a solid set of quality system essentials that limits the number of possible variables.

In contrast to blood manufacturing staff, cellular therapy staff are asked to make essentially bespoke products for every patient using a biosafety cabinet and the same expressor and centrifuge. Different products and different clinical trials have distinct donor qualifications, starting material requirements, apheresis collection parameters, processing, freezer requirements, and manufacturing steps.^36^ Different labels, reagents, and release criteria are used. Even the length of tubing segments versus the use of cryovials is non‐standard. In some cases, cleanrooms are (nonsensically) required even for closed systems. However, unlike blood component manufacturing, most traditional cellular therapy laboratories do not even have a computer system, forcing reliance on spreadsheets, paper forms and manuals, and double‐triple manual checking to identify and reduce errors.

All stakeholders in the cellular therapy space would enormously benefit from incorporating the same standardization and simplification processes found in modern blood component processing. Efforts are underway by several groups to provide some much‐needed standardization: The Alliance for Regenerative Medicine started the Standards Coordinating Body Initiative to facilitate the development of standards for the entire cell and gene therapy space, not just for cancer.^37^ ICCBBA recently published ISBT 128 Standard Labeling of Collection Products for Cellular Therapy Manufacturing (ST‐018) in 2020 and ISBT 128 Standard Chain of Identity (CoI) Identifier (ST‐028) in late 2022.^38^ Stakeholders such as the NextGen Industry Working Group and the ASTCT 80/20 taskforce^39^ are working on a tool to streamline site qualification processes performed for each vendor, utilizing a risk‐based approach to offer an abbreviated audit model for sites successfully accredited by agencies such as AABB and FACT (Foundation for the Accreditation of Cellular Therapy).

To scale effectively with current staffing levels in nursing, laboratory, and quality teams, automation is essential. The documentation, auditing, and review burdens associated with CGT workflows and handoffs are significant. Manual abstraction of chain‐of‐custody and identity elements from disparate sources is time‐consuming and unsustainable. Vendors must develop pricing models that incentivize adoption by smaller programs, which often lack resources to implement complex IT solutions.

The field must be willing to embrace standardization, automation, and IT platforms that are natively embedded with GMP principles and capable of transmitting data across the entire cell and gene therapy domain. Automating auditing, documentation, and calculations will be critical to improving efficiency in collection and processing workflows.

Questions to consider:

- Can regulatory authorities (such as FDA) work with manufacturers to allow post‐licensure standardization of non‐critical manufacturing steps, in an effort to reduce the number of product‐specific collection and processing requirements?
- Can manufacturers and clinical trial sponsors be compelled or incentivized to adopt a series of standard practices, such as collection volumes, labelling, and pre‐drug cryopreservation protocols, to diminish the burden on collection sites and laboratories to adopt unnecessary study‐specific requirements?
- Can automation be brought to traditional cell therapy laboratory processing in a cost‐efficient manner?

## 4 FROM COST CENTER TO VALUE DRIVER: REFRAMING APHERESIS IN CGT

There is perhaps no more controversial topic than that of reimbursement. Overall, the healthcare system needs to control costs, yet patients rely on the existence of a robust and growing network of collection facilities and “nearby” (a term without a consensus definition at present) high complexity laboratories to meet their needs. Both apheresis and cellular therapy laboratory work are niche activities that require the constant availability of skilled staff and an expensive infrastructure. In contrast, the current reimbursement models tend to favor paying in a lump sum and on a per‐procedure basis to a single entity, making it very difficult to explore collaborative partnerships, funding the startup costs of a new collection program, or recovering the costs of ongoing availability, especially when demand is uneven. Emerging legislation such as Texas State Bill HB3057 has begun to address patient access and cost by compelling insurance companies to cover advanced cellular therapies outside of the network of FACT accredited centers. The impact of this approach on patient access and clinical outcomes remains to be seen.

VBEs offer a legal and operational framework to support novel collaborations. Emerging from reforms to the Anti‐Kickback Statute and Stark Law, VBEs enable flexible partnerships focused on care coordination, outcome‐based payments, and shared accountability. Over 36% of US healthcare payments are now tied to alternative payment methods, including VBEs. Blood centers, as non‐profit entities, could participate in VBEs by demonstrating contributions to improved clinical outcomes, cost efficiency, and data transparency in cell and gene product delivery. As VBEs gain traction, the relationships among blood centers, hospitals, and pharmaceutical companies may shift—prioritizing patient access and equity over institutional retention and exclusivity.

In general, cellular therapy collection and laboratory activities—especially those performed in hospitals—are not reimbursed in proportion to the total cost of traditional and advanced cellular therapies. Long‐term liquid nitrogen storage, for example, is a frequently undervalued expense. When factoring in space, staffing, training and competency, equipment, ongoing maintenance, liquid nitrogen use, electricity, and monitoring, the cost to store a single bag is on the order of $100 per year. The cell and gene therapy field is expanding beyond oncology into a broad range of diseases, including rare genetic disorders (i.e., retinal dystrophies and spinal muscular atrophy); blood conditions (e.g., sickle cell disease, thalassemia, hemophilia); immune system diseases (e.g., graft‐vs‐host disease, systemic lupus erythematosis); musculoskeletal and joint disorders (e.g., osteoarthritis); and neurological disorders (e.g., Parkingson's and Huntington's diseases). These patients may be collected early in their disease course, and their products may need to be stored for future use—either periodically or after disease progression.

In collaboration with the National Association of Managed Care Physicians, the Alliance of Regenerative Medicine (ARM) published a roadmap in 2019 outlining how payers and providers can better assess the value of CGTs.^40^ ARM has also advocated modernizing CMS reimbursement policies, creating new pilot programs, ensuring Medicare and Medicaid can support access to these therapies. Among the proposed models are outcome‐based agreements, where payment is tied to a therapy's effectiveness over time; annuity‐based models that spread costs over several years; and subscription models that allow payers to access a portfolio of therapies in a “Netflix‐style” arrangement.

To alleviate financial and operational stresses, several strategies merit ongoing consideration. Reimbursement for just‐in‐case collections would allow patients to have starting material collected before a final therapeutic decision is made, increasing procedural volume and enabling more consistent availability of apheresis collections. Direct carve‐out models, where pharmaceutical companies negotiate directly with insurers, could reduce the financial burden on hospitals. Value‐based arrangements that allow patients to use Medicaid funds across state lines would improve access to specialized care. Simplified and standardized collection and manufacturing processes—supported by regulatory flexibility—could reduce costs and improve scalability. Finally, collaborative models such as VBEs offer a promising framework for cross‐institutional partnerships that prioritize patient outcomes over institutional exclusivity.

Questions to consider

- Is there a fixed percentage of the total drug cost that could be agreed upon as the standard value of a cellular therapy collection?
- Can the manufacturing success or clinical outcome be linked to the cellular collection cost?
- Can blood centers directly bill patient insurance for collection or laboratory services?

## 5 MANAGING THE WORKFORCE PIPELINE WITH TRAINING AND EDUCATION

Education and training are perhaps the ultimate examples of unrecognized, ongoing cost in healthcare. The costs are both economic (e.g., the cost to register for an activity or test) as well as time (e.g., the cost of taking an employee off a clinical/operational task so that training can occur). While educational and training programs are a pre‐requisite to the ability to offer a clinical service—and virtually every certification has some manner of continuing education requirement—the activities that lead to either initial or ongoing certification are not directly reimbursable by any payor. Some employers have sufficient scale to offer internal education sessions, which have been shown to reduce cost and improve learner satisfaction compared to external offerings.^41^ However, many employers either do not have the scale required to organize such activities or have lost the ability to offer these types of activities altogether due to the need to control costs. In these circumstances, the costs—in terms of both money and time—are typically transferred to the employee.

The COVID‐19 pandemic and its aftermath have exasperated the staffing shortage throughout healthcare, including in the clinical laboratory and the blood collection center. As discussed, this has highlighted the need for highly reliable computer systems to help with patient management, donor qualification, product handling, and regulatory compliance. The minimization of variables—such as the limitation of the number of interchangeable blood products manufactured—has helped to minimize variables, maintain affordability, and uphold quality standards. While this may limit the education and training needed to maintain a trained workforce, the reduction in autonomy that comes with automation and reduced offerings may be contributing to reduced job satisfaction.

For cellular therapy products, the pressure is perhaps in the opposite direction: the lack of standardization has led to an unclear (or, perhaps endless) product/sponsor‐specific training pathway. Product‐specific training sessions can seem endless and overwhelming for a learner. In addition, the attainment of competency to perform a task becomes more difficult to clearly establish and manage under these circumstances.^36^, ^42^ If learners begin to feel as if there is no limit on the number of training sessions that are required for interchangeable tasks, this may also lead to low job satisfaction and difficulty with retaining experienced staff.

Data from transfusion medicine fellowship programs reveals only limited enthusiasm from physicians to pursue subspecialty training in blood banking/transfusion medicine. Recent data reveal that fewer than 75% of available training slots are filled each year,^43^ a number that is viewed by many stakeholders as insufficient to justify participation in the subspecialty match offered by the National Residency Matching Program.^44^ Recently, a small number of fellowship training slots focusing on cellular therapies have been created. Participants receive an immersive experience in cellular therapy concepts but are not currently eligible for any additional subspecialty board certification.

It is unclear whether other types of providers, such as advanced practice nurses, can be recruited to fill gaps in physician coverage, especially for apheresis collections. Professional bodies such as the Advanced Practitioners Society for Hematology and Oncology and pharma companies such as Johnson & Johnson (J&J) are increasingly promoting the roles Advanced Practice Providers play in the entire patient journey, calling them “the backbone to the entire care team.”^45^ It is incumbent on the transfusion medicine physician to create effective frameworks for these providers to enhance patient access, while retaining a consequential role for physicians with subspecialty training.

In short, there is a general shortage of personnel to fill most of the slots anticipated to be required as cellular therapies begin to scale.^42^ The US Bureau of Labor Statistics (BLS) reported in 2021 that employment of medical laboratory professionals is projected to grow 11% from 2020 to 2030, faster than the average for all occupations.^46^ BLS approximates that 329,200 technologists were employed in 2021 with a shortage of up to 25 K, translating into a vacancy of 7% with an annual growth estimated at another 7%.^42^

Despite these challenges, there is broad recognition that achieving the goal of patient access to advanced therapies will require a stable and skilled workforce of many different types of contributors, including physicians, nurses, medical technologists, phlebotomists, quality specialists, and many others.^47^ One important challenge to overcome is the current economic model which all too often views education and training time as unreimbursed costs.^42^ It will be important for all stakeholders to commit the finances and time to ensure that the cellular therapy working environment is sustainable and supportive of the humans who are key to performing the needed work.

One interesting approach has been the development of certificate programs, such as the AABB Certified Advanced Biotherapies Professional and the American Society for Apheresis Qualification in Apheresis program. These programs seek to target professionals who wish to add to their skillset and reward those who complete the training with certifications. This model may prove to be more appealing to learners compared to traditional continuing education, which results in hours of credit but does not lead to a designation that may be marketable for the learner.

Ongoing development of training and education programs is essential to the ability to deliver on the promise of advanced cellular therapies. At present, the workforce is strained by a combination of factors, including: difficulty recruiting physicians and laboratory professionals to the field; content matter that is either overly proscribed (to the detriment of autonomy) or excessively non‐standardized (to the detriment of achieving and maintaining competency); and the loss of education and training activities as a result of economic pressures and lack of ability to obtain reimbursement for educational time. In order for the availability of cellular therapies to scale and meet demand, education and training must evolve to engage learners at a reasonable cost.

Questions to consider:

- Can transfusion medicine education in general—and cellular therapy topics in particular—be introduced earlier and emphasized more in the education of physicians, nurses, and laboratory staff?
- What additional professionals—such as advanced practice providers—will be needed to support expanded access and care delivery?
- Do certificate programs fill a crucial gap for learners, or do they add cost without delivering the skills necessary for participants to advance in their careers?
- Who should bear the cost of education and continuing training—employers, learners, clinical trial sponsors, payors, or other stakeholders? How can learners be incentivized or rewarded for updating their skills?
- As CGT infusions expand beyond oncology, could a new hospitalist‐like role emerge to recognize and manage cellular therapy‐related adverse events—and are TMSs uniquely positioned to fill this role?

## 6 CONCLUSION

The rapid ascension of cellular therapies to the forefront of medicine presents transfusion medicine physicians with a rare and timely opportunity to redefine their role as strategic enablers of advanced clinical biotherapies able to contribute directly to patient access and quality of care. To meet the growing demand for personalized, high‐complexity care, the field must evolve beyond traditional models and embrace scalable infrastructure, collaborative partnerships, and standardized practices that reduce cost and complexity without compromising quality. The blood center's legacy of offering low‐cost, high‐value biologically derived products from healthy volunteer donors within the strong regulatory framework created by competent authorities such as the FDA and promotion of best practices and standards by organizations such as the AABB offer a valuable blueprint. Adapting that model to bespoke cellular therapies, however, will require the transfusion medicine physician to commit to innovation, flexibility, and direct involvement in patient care.

Central to this transformation is the optimization of not only apheresis collection, but the recruitment, selection, and protection of autologous and allogenic donors of CSM. The transfusion medicine physician is well‐acquainted with their role as donor safety advocate and ethical stewardship of donated products. As donor profiling expands to include genomic, proteomic, and immunologic data, frameworks like GINA offer protection, but a more robust and transparent consenting process will evolve as the amount of data collected and analyzed exponentially increases. This includes data from assays that may not have been considered or even available when the donor was initially collected. The lack of longitudinal safety data for frequent donors—particularly those contributing 10 billion white blood cells per donation (a full leukopak)—raises serious concerns about the potential for immune depletion, compensatory clonal expansion, and other long‐term risks. Without evidence‐based guidelines and standardized monitoring, the sustainability of the donor pool may become compromised, threatening the reliability and scalability of the CSM supply.

Ultimately, the future of clinical biotherapies will depend on our ability to balance innovation with equity, efficiency with safety, and complexity with compassion. Transfusion medicine physicians are uniquely positioned to lead this charge and thereby close the gap between clinical care, laboratory science, and manufacturing logistics. By embracing their evolving role and advocating for ethical, data‐driven, and patient/donor‐focused practices, transfusion medicine specialists can help ensure that the promise of cellular therapies is realized as safely and ethically as possible.

## CONFLICT OF INTEREST STATEMENT

The authors have disclosed no conflicts of interest.

## Data Availability

Data sharing not applicable to this article as no datasets were generated or analysed during the current study.
